# Genotype–Phenotype Correlations for Putative Haploinsufficient Genes in Deletions of 6q26-q27: Report of Eight Patients and Review of Literature

**DOI:** 10.1055/s-0042-1743568

**Published:** 2022-03-11

**Authors:** Xiaolei Xie, Hongyan Chai, Autumn DiAdamo, Brittany Grommisch, Jiadi Wen, Hui Zhang, Peining Li

**Affiliations:** 1 Department of Genetics, Yale University School of Medicine, New Haven, Connecticut, United States; 2 Molecular Diagnosis Center, The Sixth Affiliated Hospital of Guangzhou Medical University, Qingyuan People's Hospital, Qingyuan, Guangdong, People's Republic of China

**Keywords:** deletions of 6q26-q27, array comparative hybridization, haploinsufficient gene, genotype–phenotype correlations

## Abstract

**Background** Cytogenomic analyses have been used to detect pathogenic copy number variants. Patients with deletions at 6q26-q27 present variable clinical features. We reported clinical and cytogenomic findings of eight unrelated patients with a deletion of 6q26-q27. A systematic review of the literature found 28 patients with a deletion of 6q26-q27 from 2010 to 2020.

**Results** For these 36 patients, the sex ratio showed equal occurrence between males and females; 29 patients (81%) had a terminal deletion and seven patients (19%) had a proximal or distal interstitial deletion. Of the 22 patients with parental studies, deletions of de novo, maternal, paternal, and bi-parental inheritance accounted for 64, 18, 14, and 4% of patients, respectively. The most common clinical findings were brain abnormalities (100%) in fetuses observed by ultrasonography followed by developmental delay and intellectual disability (81%), brain abnormalities (72%), facial dysmorphism (66%), hypotonia (63%), learning difficulty or language delay (50%), and seizures (47%) in pediatric and adult patients. Anti-epilepsy treatment showed the effect on controlling seizures in these patients. Cytogenomic mapping defined one proximal critical region at 6q26 containing the putative haploinsufficient gene *PRKN* and one distal critical region at 6q27 containing two haploinsufficient genes *DLL1* and *TBP* . Deletions involving the *PRKN* gene could associate with early-onset Parkinson disease and autism spectrum disorder; deletions involving the *DLL1* gene correlate with the 6q terminal deletion syndrome.

**Conclusion** The genotype–phenotype correlations for putative haploinsufficient genes in deletions of 6q26-q27 provided evidence for precise diagnostic interpretation, genetic counseling, and clinical management of patients with a deletion of 6q26-q27.

## Introduction

Pathogenic copy number variants (pCNVs) of interstitial or terminal deletions in the long arm of chromosome 6 are rare cytogenomic abnormalities detected in approximately 0.05% of patients with intellectual disability (ID) and developmental delay (DD). 1 Earlier studies suggested simple terminal deletions of 6q as an emerging new syndrome. 2 3 Further studies using chromosome analysis, fluorescence in situ hybridization (FISH), and array comparative hybridization (aCGH) calibrated various sizes from patients with 6q deletions and constructed a phenotypic map for deletions of 6q. 4 5 6 7 A systematic review of patients with deletions of 6q summarized the phenotypic features from 28 cases and presented a deletion map from 13 cases. 8 This map showed an association of deletions of 6q23-q25 with limb defect and cleft palate, deletions of 6q26-q27 with cardiac defects, genital hypoplasia, short neck, and retinal abnormalities, and subtelomeric or terminal deletions of 6q with ID, DD, dysmorphic features, brain anomalies, seizure, hydrocephalus, microcephaly, growth retardation, and vertebral anomalies.

In the past decade, the application of high-resolution aCGH has allowed more accurate detection of genomic coordinates and gene content from pCNVs and, thus, significantly improved the diagnostic accuracy and efficacy. 9 10 11 Here, we presented the clinical features and cytogenomic results from eight unrelated patients with different deletions at the 6q26-q27 region. We also performed a literature review to evaluate the clinical and cytogenomic findings from 28 patients with a deletion of 6q26-q27. 12 13 14 15 16 17 18 19 20 21 22 23 24 25 From this series of 36 patients, we defined genotype–phenotype correlations from prenatal to postnatal stages and two critical regions with putative haploinsufficient genes and other candidate morbid genes. These results provided evidence for diagnostic interpretation, genetic counseling, and clinical management of patients with deletions of 6q26-q27.

## Materials and Methods

### Human Subjects

Eight patients with a deletion of 6q26-q27 were diagnosed at the Yale Clinical Cytogenetics Laboratory by G-band karyotyping, FISH, and aCGH. Clinical information from the initial diagnosis to follow-up visits was evaluated. This project was categorized as a chart review retrospective case study and deemed exempt from Institutional Review Board (IRB) approval and granted waiver of consent based on the policy of the Yale University IRB.

### Karyotyping, FISH, and aCGH Analysis

Karyotyping was performed on G-band metaphases of cultured lymphocytes from peripheral blood specimens following the laboratory's standard protocols. Genomic DNA was extracted from peripheral blood lymphocytes using the Gentra Puregene Kit (Qiagen, Valencia, CA, United States). FISH tests were performed on metaphase cells using a DNA probe specific for the chromosome 6q terminal region (Abbott Inc. Des Plaines, IL, United States). aCGH was performed using Agilent SurePrint G3 Human CGH + SNP microarray (Agilent Technologies, Inc., Santa Clara, CA, United States) as previously described. 9 10 The base pair designation for detected pCNVs was based on the February 2009 Assembly of human genome (GRCh37/hg19).

### Literature Review and Data Analysis

A systematic literature review was performed for case reports with deletions of 6q26-q27 from 2010 to 2020 from PubMed using the following keywords: subtelomeric 6q deletion, 6q terminal deletion, FISH, aCGH, 6q26, 6q27, 6q26-qter, and deletion. Only patients with an isolated deletion of 6q26-q27 were selected. Familial cases with multiple affected patients were counted as one by the proband. This literature search found 14 articles with 28 patients having a deletion of 6q26-q27. 12 13 14 15 16 17 18 19 20 21 22 23 24 25 Reported patients without cytogenomic mapping for the deletion, with large deletions extending to 6q25, and compound rearrangements involving other chromosomes were excluded. 26 27

Genotype–phenotype correlations were evaluated from prenatal to postnatal stages by assessing the percentage of patients with major clinical findings (>50%) and minor symptoms (<50%). The classification of the clinical significance of terminal and interstitial deletions follows the technical standards of the American College of Medical Genetics and Genomics (ACMG) by a quantitative and evidence-based scoring. 28 Critical regions were constructed by the smallest overlapped deletions or specified fragile sites with potential candidate genes. Online Mendelian Inheritance in Man (OMIM) morbid genes ( https://www.omim.org/ ) and putative haploinsufficient genes by DECIPHER haploinsufficiency index (%HI) were selected from ClinGen using the dosage sensitivity filter for 6q26-q27 region.

## Results

### Clinical and Cytogenomic Findings from the 36 Patients

As shown in Table 1 , 36 patients and their clinical features were arranged numerically at the prenatal, perinatal, infant, pediatric, and adult stages. The clinical features and cytogenomic results of eight patients with a deletion of 6q26-q27 from Yale Clinical Cytogenetics Laboratory were described as follows.

**Table 1:** The clinical features of 36 patients with deletions of 6q26-q27

Development stages	Pts (# in Ref)	Sex (age range)	Inheritance	Size (Mb)	DD/ID	Structural brain abnormality	Facial dysmorphism	Hypotonia	Learning difficulty or language delay	Seizures	Vertebral or spinal cord malformation	Hydro- cephalus	Micro- cephaly	Joint laxity	Others
Prenatal	1 (2)	F(21 gw–TOP)	dn	9.9		+						–			
	2	F(22 gw)	mat	0.38		+						+			
	3	F(29 gw–nb)	dn	4.9		+									
	4	M(30 gw–sb)	dn	4.6		+	+						+		HK
Perinatal	5	M(20 gw–21 d)	–	0.24		+	+	+			+		+		MDKH
	6 (10)	F(20 gw–1.5 y)	pat	1.5	+	+	+			–			–		
	7 (2)	F(22 gw–5m)	pat	2		+	–						–		
	8 (1)	M(32 gw–2y)	mat	3.9	+	+	+	+	+	–			–	+	
	9 (12)	M(pn–6 y)	mat	1.2		+	+	+		+	+				
Prenatal						100%									
Infant	10 (4)	F(nb)	dn	2.2		+						+			
	11 (6)	F(nb)	dn	2.2	+	+	–	+	+	+	–	+	–	–	
	12 (TS1)	M(2 m)	–	8.93	+	+		+	+	+			+		ADHD, ASD, HL
	13 (3)	F(4 m)	dn	8.1	+	+	+	+		+	+	–	macro	–	
Pediatric	14	F(4 m–8 y)	dn	5.7	+		+	+	+		+			+	EDS
	15 (5)	F(8 m)	dn	5.65	+	+	+	+	+	+		+	macro		
	16 (TS2)	M(y)	dn	7.95	+				+	+			+		
	17(TS3)	F(2 y)	dn	1.09	+			+	+						Toe-walking, autism
	18 [7]	F(2.5 y)	–	1.75	+	+	+	+	+	–	–	–	–	–	
	19	M[6 y]	dn	8	+	+	+	–	+	–		–	+		
	20 [4]	F(6 y)	–	6.2	+	+	+	+	+	–	+	–	+	–	
	21(TS4)	M(6 y)	p/m	0.08	+		+		+		_+_				Scoliosis
	22 (5)	F(7 y)	–	6	+	+	+	+		+	_+_	+			
	23 (TS5)	M(8 y)	dn	5.24	+				+	+	+				IA, VSD, PDA, OCD, ADHD
	24 (TS6)	M(8 y)	–	0.99	+										
	25 (TS7)	(9 y)	–	9.50	+	+			+	+		+	+		Poor vision
	26	F(3–12 y)	mat	0.33	+	+	+	+	+	–		+	+		
	27 (2)	M(12.5y)	–	7	+	+	+	+		+			–	+	
	28 (3)	M(13 y)	–	7	+	+	+	+		+			–	+	
	29 (6)	M(15 y)	–	3	+	+	+	+		–		–	–	+	
	30 (1)	F(17 y)	–	2.15	+	+	+	+	+	–		–			
	31 (8)	M(18 y)	–	2	+	+	+	+		+	+				
Adult	32 (TS8)	F(23 y)	dn	6.61	+		+	+	+	+					VSD, PDA, CD, EP
	33 (4)	M(25 y)	–	6	+	+	+	+		+	+				
	34 (2)	M(25 y)	dn	5.21	–	–	–	–	–	–	–	+		–	Anosmia
	35 (7)	M(33 y)	–	2.5	+	+	+			+				+	
	36	M(29 y)	pat	0.38											EOPD
Postnatal					81%	72%	66%	63%	50%	47%	31%	22%	22%	19%	

Abbreviations: –, not present; +, present; ADHD, attention deficit hyperactivity disorder; ASD, autism spectrum disorder; CD, ciliary dyskinesia; DD, Development delay; dn, de novo; EDS, Ehlers-Danlos syndrome; EOPD, early-onset Parkinson disease; EP, episodes of psychosis; gw, gestational weeks; HK, horseshoe kidney; HL, hearing loss; IA, imperforate anus; ID, Intellectual disability; mac, macrocephaly; mat, maternal; MDKH, multicystic dysplastic kidney and hydronephrosis; nb, newborn; OCD, obsessive-compulsive disorder; p/m, bi-parental; pat, paternal; PDA, patent ductus arteriosus; sb, stillbirth; top, termination of pregnancy; VSD, ventricular septal defect.

Note: Patients (Pts) were collected from this study (TS) and published reports, and number in parenthesis () refers to the patient number in TS and the reports; patients 1, 11, 13, 15, 18, and 20 were from the report of Peddibhotla et al (2015), 20 patient 2 was from Thakur et al (2018), 22 patient 3 was from Li et al (2011), 17 patient 4 was from Valduga et al (2010), 13 patient 5 was from Puvabanditsin et al (2020), 25 patients 6, 9, 22, 27, 28, 29, 31, 33, and 35 were from Conti et al (2013), 19 patients 7 and 8 were from Wadt et al (2012), 18 patient 10 was from Dupé et al (2011), 15 patient 14 was from Mosca et al (2010), 12 patient 19 was from De Cinque et al (2017), 21 patient 26 was from Hanna et al (2019), 24 patient 30 was from Rigon et al (2011), 14 patient 34 was from Gerber et al (2011), 16 and patient 36 was from Williams et al (2018). 23

### Patient One (Listed as Patient 12 in Table 1 )

A male newborn was referred for cytogenomic tests with clinical indications of microcephaly and hearing loss. The karyotype showed a terminal deletion at 6q26, denoted as 46,XY,del(6)(q26). aCGH defined an 8.930 Mb deletion at 6q26q27 (chr6:161981583_170911240) including 36 genes from *PRKN* ( *PARK2* ) to *PDCD2* . From the age of 2 years to the present age of 20 years, follow-up examinations on the patient noted central hypotonia, peripheral hypertonia, DD/ID involving both motor and speech development, seizures, attention-deficit and hyperactivity disorder (ADHD), autism spectrum disorder (ASD), cognitive limitation, and abnormal brain MRI.

### Patient Two (16)

**Fig. 1:**
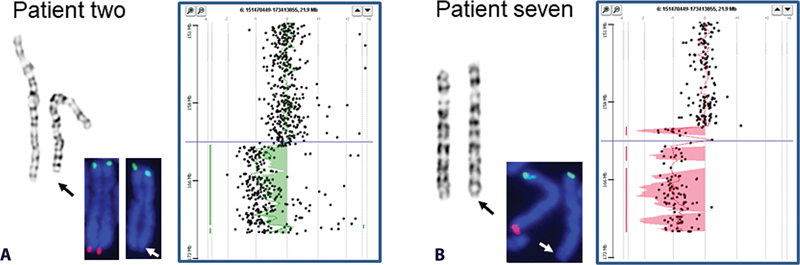
Cytogenomic results for two patients with a deletion of 6q26-q27. ( **A** ) An 8.031 Mb deletion at 6q26 in patient two (from left to right images of chromosome, FISH, and aCGH), and ( **B** ) A 9.577 Mb deletion at 6q26 in patient seven.

A 1-year-old boy was noted with microcephaly, DD, and seizures. The karyotype showed a terminal deletion at 6q26, denoted as 46,XY,del(6)(q26)dn. FISH test using subtelomeric probes for the 6p and 6q loci confirmed the terminal deletion of 6q. aCGH defined an 8.031 Mb deletion of 6q26q27 (chr6:162983518_171014790) including 36 genes from *PRKN* to *PDCD2* ( Fig. 1A ). He sat at 6 months, crawled at 13 months, pull to stand at 16 months, and took steps at 24 months. He had a significant delay in both receptive and expressive language and could say a few single words and phrases at the age of 3 years. Follow-up clinical examination up to the age of 14 years noted microcephaly, cognitive impairment, and presumably symptomatic focal impaired seizures with occasional secondary generalization. He had two surgeries to correct strabismus. Electroencephalogram performed at 9-year-old showed marked slowing over the left hemisphere and occasional left temporal sharp waves. He had been seizure-free since starting Lamictal at the age of 10 years. He was in a special education class. Chromosome analyses on both parents found normal results, indicating a de novo deletion of 6q in the affected boy.

### Patient Three (17)

A 2-year-old female had global DD, hypotonia, language impairment, toe-walking, and behavioral concerns for autism disorders. The karyotype showed a normal female karyotype, and aCGH detected a 1.089 Mb deletion at 6q27 (chr6:169818238_170906796, including nine genes *WDR27* , *PHF10* , *TCTE3* , *EMARD* [ *C6orf70* ], *DLL1* , *FAM120B* , *PSMB1* , *TBP* , and *PDCD2* ). Follow-up aCGH analysis on both parents found normal results, indicating a de novo deletion of 6q27.

### Patient Four (21)

A 6-year-old male showed global DD, speech delay, minor dysmorphic features, and scoliosis. aCGH detected an 81 kb bi-allelic deletion at 6q26 (chr6:162198305_162279506), including intron 4, exon 5, and intron 5 of the *PRKN* gene and regions of homozygosity in 1.3% of the genome. Follow-up analysis on the parents found both mother and father as heterozygous carriers for this deletion. Family history showed first cousin consanguinity in the parents.

### Patient Five (23)

Chromosome analysis on an 8-year-old boy detected a terminal deletion at 6q27, denoted as 46,XY,del(6)(q27)dn. aCGH defined a 5.244 Mb deletion at 6q27 (chr6:165667663_170911240) including 33 genes from *C6orf118* to *PDCD2* . This preterm newborn baby had an imperforate anus, which was repaired after birth. The boy was followed up from the ages of 6 to 8 years with seizures, myelomeningocele, spinal bifida, urinary incontinence, ID, obsessive-compulsive disorder (OCD), ADHD, and sleep disturbance. He also had ventricular septal defect (VSD), patent ductus arteriosus (PDA), and tethered cord with filum terminale lipoma with conus at L2 and L3 levels, and MRI findings concerned for occult dysraphism at S1/S2. Chromosome and aCGH analyses on both parents found normal results, indicating a de novo deletion of 6q in the affected boy.

### Patient Six (24)

The karyotype on an 8-year-old boy with DD showed a normal male pattern and aCGH detected a 0.985 Mb deletion involving three genes *PRKN* ( *PARK2* ), *PACRG* , and *QKI* at 6q26 (chr6:162870169_163855503) with breakpoints at intron 1 of the *PRKN* and *QKI* genes.

### Patient Seven (25)

A 9-year-old girl was noted with ID, epilepsy, microcephaly, and poor vision with corrective lenses. The karyotype showed a terminal deletion at 6q26, denoted as 46,XX,del(6)(q26). FISH test using subtelomeric probes for the 6p and 6q loci confirmed the terminal deletion of 6q, and aCGH defined a 9.577 Mb deletion of 6q26q27 (chr6:161437468_171014790) including 38 genes from *MAP3K4* to *PDCD2* ( Fig. 1B ). This patient showed congenital hydrocephalus at birth and had undergone ventriculoperitoneal shunt and endoscope. She had developmental and speech delays. She had a history of seizures and had been seizure-free on phenobarbital. The girl had early pubertal development including secondary sexual characteristics at the age of 11 years. She had difficulty in learning and language expression and received special education. She could read letters and numbers and did simple arithmetic but could not read at the age of 13 years. She received Depokote to control bleeding during menses since the age of 17 years. Follow-up clinical examinations up to the age of 21 years noted kyphoscoliosis and menorrhagia.

### Patient Eight (32)

A 23-year-old female had seizure disorder and ID (IQ < 70). She was also known to have anxiety disorder with episodes of psychosis and ciliary dyskinesia with frequent pulmonary infections. Other clinical findings included strabismus, gastroesophageal reflux, hypotonia, VSD, and PDA which resolved spontaneously. The karyotype showed a terminal deletion at 6q26, denoted as 46,XX,del(6)(q26)dn. aCGH detected a 6.608 Mb deletion at 6q26q27 (chr6:164312707_170921089) including 33 genes from *C6orf118* to *PDCD2* . Both parents had chromosome analysis done with normal results.

### Clinical Findings for Deletions of 6q26-q27

**Fig. 2:**
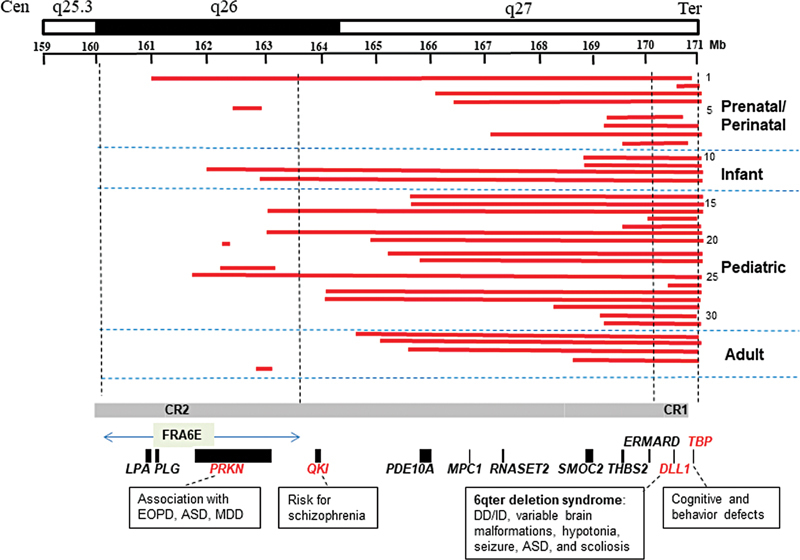
Cytogenomic mapping of critical regions and genotype–phenotype correlations for morbid genes of 6q26-q27. A cytogenomic map for sizes of deletions, critical regions, and candidate genes of 6q26-q27. The upper panel shows the chromosome 6q26-q27 region with a genomic coordinate. The middle panel shows the size and location of the 36 deletions following prenatal to postnatal stages (each red bar for a deletion and numbering on the right side matches patient number in Table 1 ). The lower panel shows critical regions (CR), morbid genes (putative haploinsufficient genes in red), and genotype–phenotype correlations for morbid genes in the 6q26-q27 region.

Of these 36 patients with deletions of 6q26-q27 ( Table 1 ), 18 were male and 18 were female; 29 patients (81%) had a terminal deletion, whereas seven patients (19%) had a proximal or distal interstitial deletion. Of the 22 patients with follow-up parental studies, 14 (64%) were de novo, four (18%) were maternally inherited, three (14%) were paternal, and one (4%) was inherited from both parents. Cytogenomic and clinical findings grouped into prenatal, perinatal, infant, pediatric, and adult stages are summarized in Fig. 2 and Table 1 . All four prenatal patients and five perinatal patients showed brain abnormalities (100%, nine out of nine) by ultrasound examination. The major postnatal clinical features observed in the five perinatal and 27 postnatal patients were DD/ID (81%, 26 out of 32), brain abnormalities (72%, 23 out of 32), facial dysmorphism (66%, 21 out of 32), hypotonia (63%, 20 out of 32), learning difficulty or language delay (50%, 16 out of 32), and seizure (47%, 15 out of 32). Other clinical features included vertebral or spinal cord malformation (31%, 10 out of 32), microcephaly (22%, 7 out of 32), hydrocephalus (22%, 7 out of 32), joint laxity (19%, 6 out of 32), VSD, ADHD, and ASD (6%, 2 out of 32), as well as hearing loss, poor vision, and early-onset Parkinson disease (EOPD) (3%, 1 out of 32).

### Critical Regions and Genotype–Phenotype Correlations within 6q26-q27

Four patients with a small distal interstitial deletion (patients 6, 9, 17, and 31 in Table 1 ) were mapped critical region 1 (CR1) spanning 1.1 Mb, which encompassed the *WDR27* , *PHF10* , *TCTE3* , *ERMARD* , *DLL1* , *FAM120B* , *PSMB1* , *TBP* , and *PDCD2* genes. 19 The CR1 contained haploinsufficient genes for deletions of 6q27. Four proximal interstitial deletions at 6q26 involving the *PRKN* gene were noted in two patients with DD, a preterm neonate, and an adult with EOPD (patients 5, 21, 24, and 36 in Table 1 ). 23 25 Following the ACMG guidelines for CNV classification, these interstitial deletions were scored 0.15 to 0.3 and were considered as a variant of uncertain significance (VUS). 28 Excluding pseudogenes, there were 53 coding genes in the 6q26-q27 region. Of them, 11 OMIM morbid genes *LPA* , *PLG* , *PRKN* , *PDE10A* , *MPC1* , *RNASET2* , *SMOC2* , *THBS2* , *ERMARD* , *DLL1* , and *TBP* , three putative haploinsufficient genes *PRKN* , *QKI* , and *TBP* (by DECIPHER HI index <10%), and one haploinsufficient gene *DLL1* (by HI score and HI index in ClinGen) were listed as candidate genes. The fragile site at 6q26, FRA6E, spanned approximately 3.6 Mb (chr6:160000000_163600000). 29 This region, containing the putative haploinsufficient gene *PRKN* and breakpoints of four interstitial and six terminal deletions, was defined as critical region 2 (CR2). These two critical regions with known morbid and haploinsufficient genes of 6q26-q27 and genotype-phenotype correlations are shown in Fig. 2 .

## Discussion

From the 36 patients with a deletion of 6q26-q27, the equal ratio of male versus female (50 vs. 50%) showed no gender difference. Approximately, two-thirds of these 6q deletions were de novo and one-third were inherited, indicating that a significant portion was familial; thus, follow-up parental study was recommended. Patients with a deletion at 6q27 showed scattered breakpoints with different sizes and the lack of recurrent deletions. The lack of clusters of segmental duplications or low copy repeats at 6q26-q27 region was noted from the Human Genome Browser ( http://genome.ucsc.edu/ ) and likely explained the absence of recurrent deletions caused by non-allelic homologous recombination. Therefore, the deletion formation mechanism most likely involved the repair of subtelomeric double-strand breaks at 6q27. However, the breakpoints at 6q26 fell in the FRA6E site and suggested a “hotspot” for interstitial and terminal deletions. 29 Further, sequence analysis of breakpoints at 6q26 could define the underlying mechanism of these deletions and their relations to the FRA6E site.

A recent retrospective analysis of cytogenomic abnormalities detected by aCGH showed a detection rate of subtelomeric pCNVs in 1% of pediatric patients and 0.5% of prenatal cases. 11 For the 36 patients with a deletion of 6q26-q27, nine were detected prenatally, 22 were detected in infancy and childhood, and five were detected in adulthood. These results indicated that over two-thirds of patients were detected postnatally with variability in onset and severity of diseases. Of the nine fetuses detected prenatally, termination of pregnancy was decided for a fetus with a 9.9 Mb deletion, stillbirth was noted in a fetus with a 4.6 Mb deletion, and the remaining fetuses had deletions in the size ranging from 0.38 to 4.9 Mb. These observations suggested an increased risk of stillbirth for terminal deletions of 6q in prenatal diagnosis.

An approach combining cytogenomic mapping and bioinformatic mining has been used to define critical regions and interacting candidate genes for ID. 30 The collection of more patients and the curated databases with improved bioinformatic tools enabled precise genotype–phenotype correlations and fine mapping of critical regions with haploinsufficient genes. The CR1 at 6q27 contained three morbid genes *ERMARD* (OMIM615544), *DLL1* (OMIM618709), and *TBP* (OMIM607136), and the last two were haploinsufficient. The clinical findings from two patients (patients 2 and 26 in Table 1 ) with a smaller deletion including the *DLL1* and *TBP* genes but not the *ERMARD* gene indicated that haploinsufficiency of these two genes is sufficient to cause the 6q terminal deletion syndrome. 22 24 Recent exome sequencing in patients with neurodevelopmental disorders identified heterozygous pathogenic variants in the *DLL1* gene in 12 unrelated families; the most common clinical features for these patients were ID, ASD, seizures, variable brain malformations, muscular hypotonia, and scoliosis. 31 Similar clinical features were noted in patients with a deletion involving the CR1 at 6q27. These clinical and genetic findings support haploinsufficiency of the *DLL1* gene as a mechanism for the pathogenesis of the 6q terminal deletion syndrome. The *DLL1* gene encodes a human homolog of a Notch Delta ligand involved in cell adhesion, cell communication and fate determination, and Notch pathway of biological processes. The *DLL1* protein played a major role in the nervous system of paraxial mesoderm during somitogenesis; mice with *DLL1* deletion showed severe nervous system defects in embryogenesis. 15 32

The *TBP* gene encodes a TATA-binding protein involved in the initiation of transcription by forming transcription complex TFIID associated with TBP-associated factors. This transcription complex regulates many biological processes of neurodevelopment disorders. The CAG repeat expansion in the N-terminal domain of *TBP* causes late-onset spinocerebellar ataxia. 33 One patient analyzed by BAC clone FISH showed a smaller terminal deletion of approximately 400 kb involving only the *PSMB1* , *PDCD2,* and *TBP* genes. 4 The study of heterozygous TBP mice suggested that *TBP* is potentially involved in cognitive development. 7 A functional study noted that the silencing of *ERMARD* in the developing rat neocortex produced periventricular nodular heterotopia. 14 These results implied modifying effects from the *TBP* and *ERMARD* genes for the 6q terminal deletion syndrome. Additionally, intra-family variability was noted in a phenotypically normal carrier mother with a terminal deletion of 0.6 to 0.8 Mb at 6q27 and two pregnancies of a malformed fetus. 34 Furthermore, large deletions extending to 6q25, compound rearrangements involving 6q27 and other chromosomes, and ring chromosome 6 involving a deletion of 6q27 also present clinical features of the 6q terminal syndrome. 26 27 35

The *PRKN* gene encodes a RING domain-containing E3 ubiquitin ligase involved in proteasome-dependent degradation of proteins, and pathogenic variants in the gene are known to cause Parkinson's disease (OMIM600116). A 381 kb deletion including exons 1 and 2 of the *PRKN* gene was transmitted from an unaffected father to his son; an additional de novo 71 kb deletion in trans involving exon 3 of the *PRKN* gene in the son likely contributed to the EOPD with the onset of disease at the age of 20 years. 23 CNVs affecting different exons 2 to 7 of the *PKRN* gene have been found in familial cases with asymptomatic carriers, high-function autism, and ASD. 36 A recent study indicated a significant association of short deletions near the *PRKN* gene with a major depressive disorder (MDD). 37 These studies indicated the associations of intra-genic *PRKN* deletions with EOPD, ASD, and MDD. Even though small deletions involving the *PRKN* gene are most likely classified as VUS by current technical standards, clinical re-evaluation of psychologic and behavior disorders in follow-up visits and laboratory re-analysis of the deletion and its impact on gene function should be considered. A patient with clinical features of the 6q terminal deletion was detected with a reciprocal balanced translocation, t(5;6)(q23.1;q26); the breakpoint at 6q26 disrupted the *QKI* gene and decreased its expression. 38 The *QKI* gene belongs to a family of RNA-binding protein with an HNRNPK homology and KH domain, which regulates RNA splicing, export of target RNAs from nucleus, translation of proteins, and RNA stability; its regional specificity to target genes in the human prefrontal cortex and hippocampus has made it a candidate gene for schizophrenia. 39 The clinical significance for CR2 requires further investigation from more patients with deletions involving the *PRKN* and *QKI* genes.

## Conclusions

We described eight unrelated patients and reviewed 28 patients with a deletion of 6q26-q27 to summarize the clinical features at fetal, infant, pediatric, and adult stages. The estimated occurrence of major and minor clinical features provided evidence for reduced penetrance and variable expressivity for patients with a deletion of 6q26-q27. One proximal critical region CR2 at 6q26 and one distal critical region CR1 at 6q27 were defined. Haploinsufficiency of the *DLL1* gene at CR1 explained the pathogenesis of the 6q terminal deletion syndrome. Effects of the *TBP* and *ERMARD* genes on CR1 require further investigation. The deletions involving the truncation and intra-genic loss of exons in the *PRKN* gene at CR2 may associate with EOPD, ASD, and MDD. Disruption of the *QKI* gene may associate with schizophrenia. The reporting for deletions of 6q26-q27 should suggest the increased risk of prenatal stillbirth and postnatal psychologic and behavior disorders. Further clinical evaluation on more patients and genetic analysis to define the gene disruption and functional deficiency is needed to understand the reduced penetrance, variable expressivity, and modifying effects for deletions of 6q26-q27.
